# Development of a MEMS Piezoresistive High-g Accelerometer with a Cross-Center Block Structure and Reliable Electrode

**DOI:** 10.3390/s24175540

**Published:** 2024-08-27

**Authors:** Cun Li, Ran Zhang, Le Hao, Yulong Zhao

**Affiliations:** State Key Laboratory for Manufacturing Systems Engineering, Xi’an Jiaotong University, Xi’an 710049, China; cun.li@xjtu.edu.cn (C.L.); hlxidian@163.com (L.H.); zhaoyulong@xjtu.edu.cn (Y.Z.)

**Keywords:** high-overload accelerometer, center block, piezoresistive, ohmic contact

## Abstract

A MEMS piezoresistive sensor for measuring accelerations greater than 100,000 g (about 10^6^ m/s^2^) is described in this work. To enhance the performance of the sensor, specifically widening its measurement range and natural frequency, a cross-beam construction with a center block was devised, and a Wheatstone bridge was formed by placing four piezoresistors at the ends of the fixed beams to convert acceleration into electricity. The location of the varistor was determined using the finite element approach, which yielded the optimal sensitivity. Additionally, a reliable Pt-Ti-Pt-Au electrode was designed to solve the issue of the electrode failing under high impact and enhancing the stability of the ohmic contact. The accelerometer was fabricated using MEMS technology, and the experiment with a Hopkinson pressure bar and hammering was conducted, and the bias stability was measured. It had a sensitivity of 1.06 μV/g with good linearity. The simulated natural frequency was 633 kHz The test result revealed that the accelerometer can successfully measure an acceleration of 100,000 g.

## 1. Introduction

MEMS accelerometers are a new type of sensor fabricated using microelectromechanical system technology. Compared to conventional accelerometers, they are smaller and lighter, and consume less power [1]. Accelerometers are widely used in various industries, including automotive, biomedical, consumer electronics, robotics, and military applications. In some cases, these devices are subjected to shock overloads of tens or even hundreds of thousands of g [2,3].

Due to the harsh working environment of high-g accelerometers, these sensors require a very large measurement range and high sensitivity [4,5]. Although high sensitivity and a large measurement range are typically mutually exclusive, they both can be enhanced to a certain extent through innovative structure design. Yunbo Shi et al. proposed a beam and center island mass silicon structure with an experimental sensitivity of 0.5611 μV/g [6]. The procedure is a little bit difficult since the proof mass structure must be etched several times throughout the processing. Chen Jia et al. proposed a self-supported sensing beam accelerometer with a measurement sensitivity of 0.54 μV/g/V and a resonant frequency of 445 kHz [7]. This construction somewhat reduces the overload capability. A new piezoresistive accelerometer with overload resistance characteristics based on a membrane island structure was proposed by Jie Zhang et al. Three prototypes were tested with sensitivities of 0.579, 0.505, and 0.532 μV/g, respectively, and a maximum peak sensitivity repeatability of 11%. The range was up to 200,000 g and the overload ability reached up to 250,000 g [8]. This structure has the advantage of having an ultra-high range, but the sensitivity decreased slightly. A silicon micromechanical high-impact accelerometer with a bonded hinge structure was proposed by Kebin Fan et al. Their shock test results showed that the sensitivity of the accelerometer was 0.516 μV/g at a shock acceleration of 44,614 g under a 5 V excitation [9]. This structure is novel but may lead to an increase in the linearity error of the sensor. Xiaodong Hu et al. proposed a high-acceleration sensor with a double-clamped beam carrying a transversal and a longitudinal piezoresistor on each end of the beam [10]. This structure will have a little lower sensitivity, but it is clear and straightforward. A MEMS accelerometer with a self-supported piezoresistive element was presented by Robert Kuells et al. It can withstand shocks of 75,000 g with a sensitivity range of 0.035 to 0.23 μV/V/g and an intrinsic frequency range of 2.7 to 3.7 MHz [11].

Most of the sensors mentioned above are piezoresistive high-g accelerometers. Piezoresistive high-g accelerometers are a common type of high-g-value accelerometers, often found in military equipment, and are significant for national security [12]. For these applications, these sensors must operate in extreme stress environments ranging from 100,000 to 200,000 g [13,14]. The accelerometer has no conventional proof mass, and as a substitute, the cross-beam plays the role of proof mass and support, which optimizes the stress concentration and can bear the impact of higher accelerations with higher natural frequencies. The cross-beam has a uniform thickness, and at the junction point of the beam, the width is enlarged to increase its weight for higher sensitivity. Therefore, the piezoresistive high g-value accelerometer developed in this study can improve sensitivity and the measurement range, and can also reduce manufacturing complexities. Additionally, the ohmic contact area in the lead portion was also enhanced, resulting in more stable signal outputs under high loads.

In summary, this paper presents the development of an accelerometer based on a cross-beam with a center block structure. The sensor model underwent theoretical analyses, and its dimensions were optimized. The finite element method was used to analyze the stress–strain relationship and natural frequency of the sensor chip after establishing the structural dimensions. Additionally, the fabrication and testing of the MEMS accelerometer are discussed. The accelerometer had a measurement range exceeding 100,000 g (g = 9.8 m/s^2^) and a natural frequency of 633 kHz.

## 2. Structure Design and Optimization

### 2.1. Structure Design

**Figure 1:**
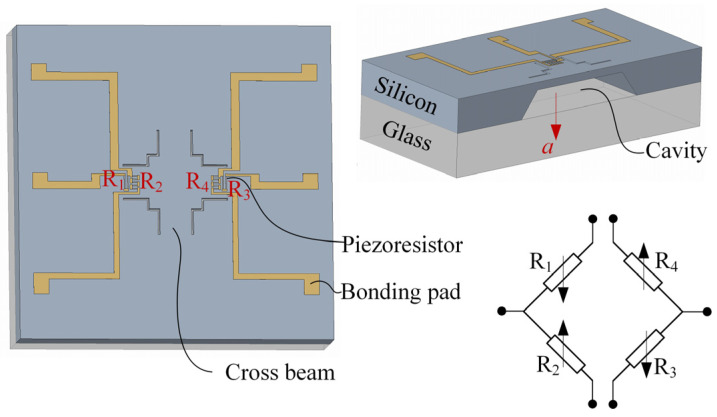
Model of sensor.

The acceleration sensor chip, utilizing a unique cross-beam structure, is illustrated in Figure 1. This design integrates the high sensitivity of a beam structure with the high impact resistance of a membrane structure. Notably, the entire beam maintains a uniform thickness without incorporating a proof mass, enhancing the sensor’s natural frequency and impact resistance. Additionally, to increase the sensor’s sensitivity, the size of the supported ends of the beams is smaller than the middle region at the intersection of the four beams, focusing stress in the beam. As stress increases, the sensitivity also improves.

### 2.2. Theoretical Analysis

**Figure 2:**
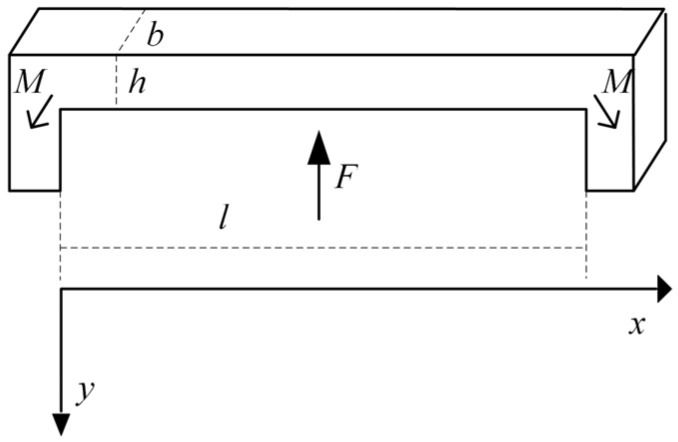
Simplified double-ended solidly supported beam.

After completing the structural model of the sensor chip, it is essential to analyze the key parameters of the sensing beam, including strain, stress, and natural frequency. Since the design was based on a standard four-beam structure, a direct quantitative formulaic analysis was not feasible. However, due to the structure’s rotationally symmetric features within the plane, it can theoretically be reduced to a double-ended fixed-support beam. The simplified force analysis of a double-ended solidly supported beam is illustrated in Figure 2.

The double-ended fixed-support beam is a part of a three-dimensional superstatic system, the fixed-end bending moment equation is

(1) $$M_{0}=\frac{1}{12}mal$$

where *M*_0_ is the fixed end bending moment (N∙m); *l* is the length of the beam (m); *F* is the solid end support (N); *m* is the mass of the beam (kg); and *a* is the acceleration (m∙s^−2^).

For any interface of a double-ended solidly supported beam, calculating the shear force and bending moment of the section yields

(2) $$\left\{\begin{array}{l} F_{x}=\frac{ma}{2l}\left(l-2x\right)\\ M_{0}+M_{x}-F_{x}x-\frac{max^{2}}{2l}=0 \end{array}\right.$$

where *F_x_* is the shear force at the section (N) and *M_x_* is the bending moment at the section (N∙m).

The bending moment at any cross-section can be obtained by rearranging Equation (2) to

(3) $$M(x)=-\frac{ma}{2l}x^{2}+\frac{ma}{2}x-\frac{mal}{12}$$

From the moment at any cross-section (Equation (3)), the maximum moment of the beam is at the solidly supported end of the beam can be calculated as

(4) $$M_{\max}=\frac{mal}{12}$$

According to the formula for the bending positive stress in the cross-section of the beam, the bending positive stress in any cross-section is calculated as

(5) $$\sigma =\frac{M}{W}$$

where *σ* is the positive bending stress in the beam section (Pa) and *W* is the flexural shear modulus (m^3^).

The flexural shear modulus of the beam can be calculated using the following equation:

(6) $$W=\frac{I}{h/2}=\frac{bh^{3}/12}{h/2}=\frac{bh^{2}}{6}$$

where *I* is the moment of inertia of the cross-section of the beam (m^4^); *b* is the beam width (m); and *h* is the beam thickness (m).

Bringing Equation (6) into Equation (5), the bending positive stress and maximum bending stress in any section of the beam is

(7) $$\left\{\begin{array}{l} \sigma =\frac{3ma}{bh^{2}}(-\frac{1}{l}x^{2}+x-\frac{l}{6})\\ \sigma_{\max}=\frac{mal}{2bh^{2}}=\frac{\rho al^{2}}{2h} \end{array}\right.$$

where *ρ* is the material’s density (kg∙m^−3^).

It is known from the calculations that the stresses are maximal at the ends of the double-ended solidly supported beam, and the following calculations are used for the deformation at any point on the beam.

According to the differential equation for the deflection of a double-ended solidly supported beam:

(8) $$EIy\prime \prime =-M(x)=\frac{ma}{2l}x^{2}-\frac{ma}{2}x+\frac{mal}{12}$$

where *y*″ is the deflection of the beam (m) and *E* is the modulus of elasticity (Pa).

Integrating Equation (8) yields

(9) $$\left\{\begin{array}{l} EIy\prime =ma(\frac{1}{6l}x^{3}-\frac{1}{4}x^{2}+\frac{l}{12}x)+C\\ EIy=ma(\frac{1}{24l}x^{4}-\frac{1}{12}x^{3}+\frac{l}{24}x^{2})+Cx+D \end{array}\right.$$

where *C* and *D* are integral term coefficients that are to be determined.

According to the solidly supported beam boundary condition and continuity condition:

(10) EIy(0)=EIy(l)=0

Bringing Equation (10) into Equation (9) yields

(11) $$\left\{\begin{array}{l} C=0\\ D=0 \end{array}\right.$$

Thus, the deflection equation for the entire double-ended solidly supported beam structure is given by

(12) $$y=\frac{ma}{12EI}(\frac{1}{2l}x^{4}-x^{3}+\frac{l}{2}x^{2})$$

where *m* is the overall quality of the beam (kg); *a* is the acceleration (m∙s^−2^); *I* is the moment of inertia of the cross-section of the beam (m^4^); and *E* is the modulus of elasticity (Pa).

When *x* = l/2, the deflection of the beam is maximal at the midpoint of the beam.

(13) $$y_{\max}=y(\frac{l}{2})=\frac{mal^{3}}{32Ebh^{3}}=\frac{\rho al^{4}}{32Eh^{2}}$$

where *b* is the beam width (m); *h* is the beam thickness (m); and *ρ* is the material’s density (kg∙m^−3^).

When examining the stress–strain relationship in the four-beam structure, it is essential to consider the correction factor. The correction factor is approximately equal to the ratio of the mass of the four-beam structure to the mass of the two solidly supported beams. Based on calculations, the correction factor is roughly 0.8. This indicates that the maximum stress on and deformation of the beams of the four-beam structure are reduced compared to a similar two-beam setup:

(14) $$\left\{\begin{array}{l} \sigma_{\max}^{\prime }=\frac{4}{5}\sigma_{\max}=\frac{2\rho al^{2}}{5h}\\ y_{\max}^{\prime }=\frac{4}{5}y_{\max}=\frac{\rho al^{4}}{40Eh^{2}} \end{array}\right.$$

When analyzing the intrinsic frequency, the sensing structure is also simplified to a double-ended solidly supported beam. To facilitate calculations and closely match the deflection curves, we based the analysis on the vibration pattern of a double-ended solidly supported beam during vibration. We assumed the following vibration mode function:

(15) $$y=\frac{y_{0}}{2}(1-\cos(\frac{2\pi }{l}x))$$

According to the Rayleigh–Ritz method, the natural frequency is

(16) $$f=\frac{\omega }{2\pi }=\frac{h\pi }{3l^{2}}\sqrt{\frac{E}{\rho }}$$

The first-order mode shapes of the four-beam structure closely resemble those of double-ended supported beam structures. Therefore, when modifying the formula to calculate the natural frequency of the four-beam structure, the correction coefficient can be assumed to be 1. This implies that the natural frequency remains unchanged.

**Figure 3:**
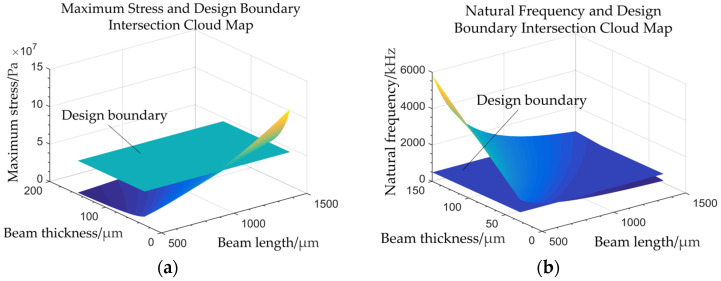
(**a**) Maximum stress and design boundary intersection cloud diagram; (**b**) natural frequency and design boundary intersection cloud diagram.

The stress–strain characteristics and natural frequency of the sensor under acceleration shock are influenced by the length and thickness of the chip’s sensing structure, as well as the chosen fabrication material and the acceleration value itself. After selecting silicon as the material and establishing the design parameters of the sensor, the relationship between the length and thickness of the sensing structure, the maximum stress and natural frequency of the structure under an acceleration impact of up to 100,000 g was analyzed. The results are depicted in Figure 3. As can be observed, an increase in the length of the sensing beam structure led to a rise in the maximum stress and a reduction in the natural frequency. Conversely, an increase in beam thickness resulted in a decreased maximum stress and elevated natural frequency. Since the maximum stress on the sensing beam directly influences sensor sensitivity, the natural frequency and sensor sensitivity are inherently conflicting. Therefore, the design of the sensing structure must strike a balance to meet the required design guidelines.

Following the principle of linearity in sensor design guidelines and to maintain the output linearity, the maximum strain in the sensitive structure should not exceed 500 με (με = 10^−6^ ε, where ε is the ratio of the deformation to the original size) [15]. To ensure sensor safety during overload conditions, the strain range of the sensor during its maximum operational range was set to between 350 and 500 με. Using the stress formula, *σ* = *Eε* and referencing the elastic modulus parameters for silicon in the (100) crystal orientation, the permissible stress range for the sensor was calculated to be between 46 *MPa* and 65 MPa. Additionally, considering the requirement for a high natural frequency, the natural frequency of the sensor chip must exceed 500 kHz. Consequently, Figure 3 illustrates the stress and natural frequency cloud diagrams and their intersections with design boundaries across various sensitive structure sizes.

**Figure 4:**
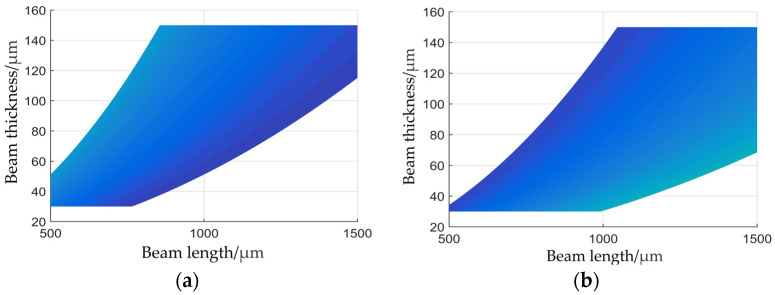
(**a**) Dimensions to meet stress requirements; (**b**) dimensions to meet natural frequency requirements.

To more clearly visualize the range of dimensional parameters for the sensing structure, the parameter range meeting the design criteria in Figure 3 was mapped onto a planar coordinate system, as depicted in Figure 4.

Based on the structural parameter range that complies with the design guidelines shown in Figure 4 and considering the overall design parameters of the acceleration sensor described in this article, as well as laboratory conditions, the final sensor chip dimensions were set at 3 mm × 3 mm × 0.85 mm. The composite beam was 1000 μm × 300 μm × 80 μm, while the center block was 500 μm × 500 μm × 80 μm. Substituting the determined structural dimensions into Equations (14) and (16) indicates that the sensor chip’s maximum stress under an acceleration of 150,000 g is 17.13 MPa, and its natural frequency is 625 kHz, both of which comply with the design guidelines.

### 2.3. Static and Modal Analysis

**Figure 5:**
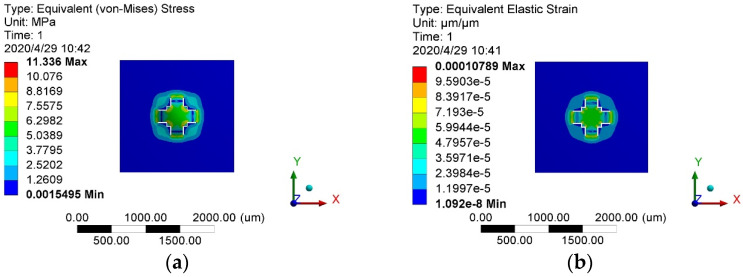
Stress–strain simulation cloud at 100,000 g: (**a**) stress map; (**b**) strain cloud diagram.

The complete modeling of the sensor chip structure with the specified dimensions was analyzed using the finite element method. Constraints and loads were applied, with acceleration loads set at 100,000 g. After solving the model, the stress–strain distributions under the specified acceleration impacts were visualized, as shown in Figure 5.

**Figure 6:**
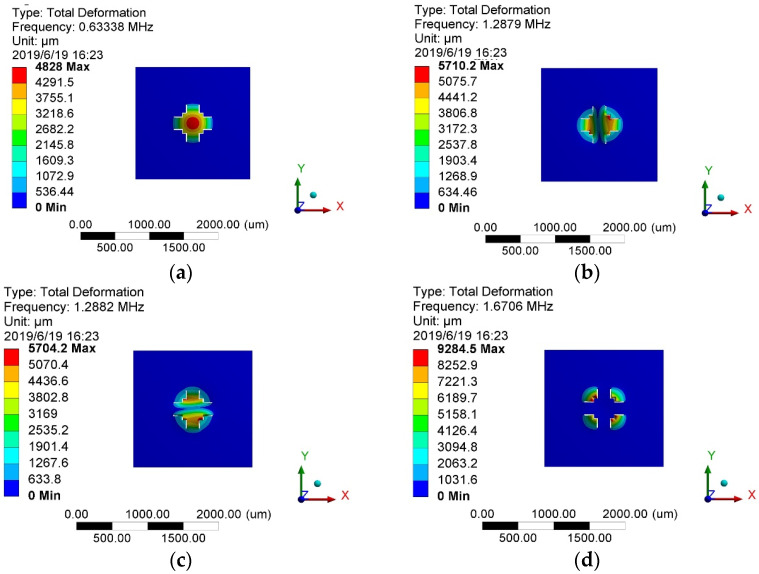
Sensor chip modal simulation cloud of each order: (**a**) first-order mode; (**b**) second-order mode; (**c**) third-order mode; (**d**) fourth-order mode.

The modal analysis primarily identified the vibration frequencies of each mode for the sensor chip. This analysis helps prevent sensor resonance caused by external vibrations, which could potentially damage the sensor. Serving as the foundation for the dynamic analysis, the modal analysis revealed the first four modes, as depicted in Figure 6.

For the sensor discussed in this study, resonance can occur in the sensing structure when the acceleration signal frequency approaches the sensor’s operating frequency. This phenomenon can damage the beam structure, leading to sensor failure. Therefore, to ensure proper functionality, the sensor’s operating frequency should significantly exceed the acceleration signal frequency. According to Figure 6, the first-order mode represents the sensor’s working mode, with a vibration frequency of 633 kHz. The intrinsic frequency is close to the value obtained through analysis (625 kHz). Furthermore, the substantial gap between its first-order vibration frequency and the frequencies of higher-order modes ensures the sensor’s safe operation under high-frequency acceleration signals.

### 2.4. Arrangement of Piezoresistors

**Figure 7:**
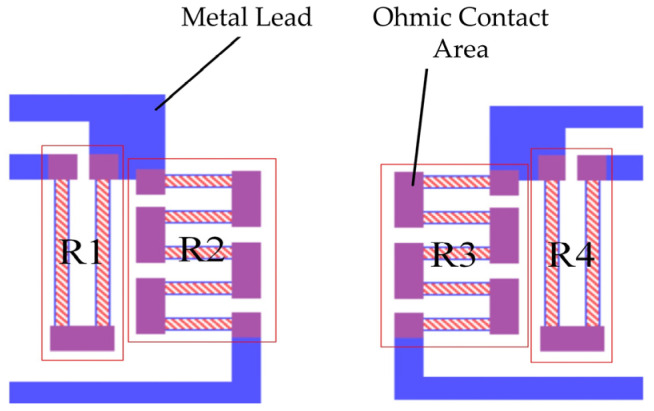
Resistor arrangement.

To prevent unequal stress caused by an excessively long resistor, a multi-fold-type resistor was utilized. Considering process precision, the resistor’s width was ultimately set at 10 μm, with a specific distance maintained between each fold. To minimize inaccuracies caused by the reverse piezoresistive effect at the connections, heavily doped silicon and ohmic contacts were employed between each pair of resistor folds. The arrangement of the resistors is shown in Figure 7.

To determine the optimal arrangement of the piezoresistor on the beam, the stress distribution along the sensor’s line of symmetry was simulated under a 100,000 g acceleration force.

**Figure 8:**
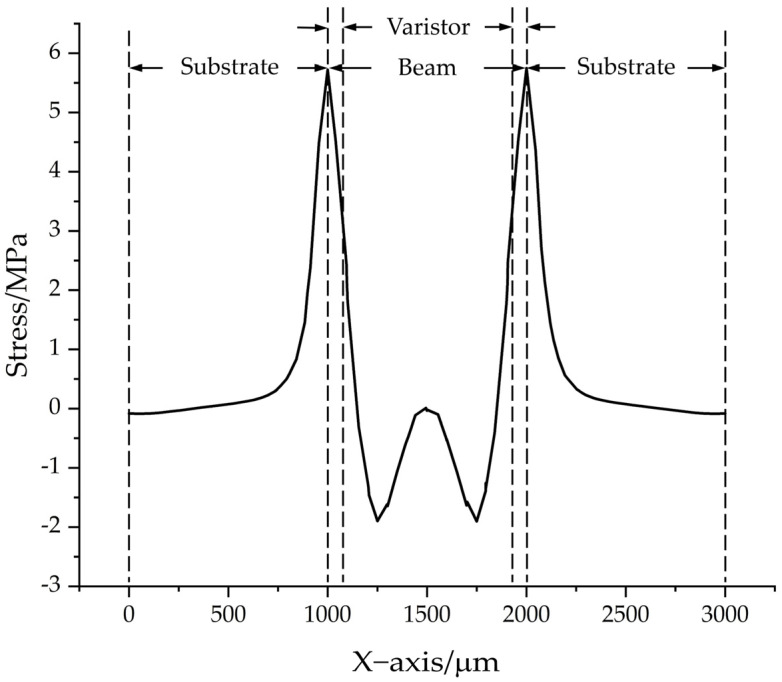
Stress profile on the upper surface of the chip.

The resulting stress distribution curve is depicted in Figure 8. It is evident that higher stress occurred at the solidly supported end of the beam. Therefore, placing a piezoresistor in the designated area, as indicated in the figure, can effectively utilize the stress variation in the beam to enhance sensor sensitivity. This suggests that the specified piezoresistor arrangement zone can fully exploit the beam’s stress fluctuations, thereby amplifying sensor responsiveness.

## 3. Fabrication, Package, and Improved Electrodes

### 3.1. Fabrication

**Figure 9:**
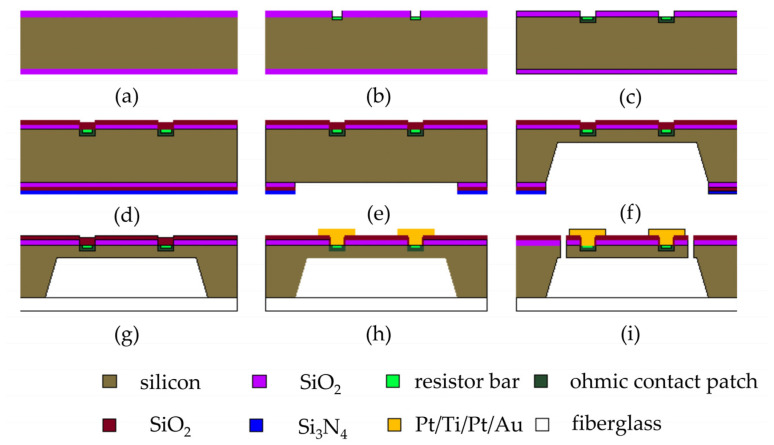
Process flow diagram of acceleration sensor chip.

The fabrication process of the sensor was based on micromachine technology. The fabrication process of the acceleration sensor chip is illustrated in Figure 9. The specific steps of the process are as follows:

(a) Prepare and cleanse the chip;
(b) Perform photolithography and patterning of the piezoresistor, and then etch the silica layer in the patterned region, followed by light doping;
(c) Execute photolithography in the ohmic contact area and proceed with heavy doping;
(d) Conduct rapid annealing to activate the introduced elements, and deposit the SiO_2_ layer as an insulating layer on the prototype’s surface using the PECVD technique;
(e) Engage in etching of the design in the back cavity area and etch the SiO_2_ layer within the graphic region;
(f) Protect the chip’s front side with a specialized fixture for wet etching, then wet etch the back cavity, ensuring that the thickness aligns with the design value;
(g) Protect the front of the sample, eradicate the SiO_2_ layer from the chip’s backside, and employ the silicon-glass anode bonding process to fuse the silicon and glass;
(h) Perform lithography in the lead hole region, corrode the silicon dioxide layer’s graphic region, deposit Pt metal through the sputtering process, and strip to reveal the lead hole with the deposited Pt layer. Utilize a tube furnace for high-temperature annealing of the prototype, establishing a local ohmic contact inside the lead hole. Execute photolithography of the metal lead region, deposit the metal Ti/Pt/Au onto the prototype’s surface through magnetron sputtering, and strip to display the metal lead;
(i) Perform photolithography on the beam and release holes, and then etch the release holes utilizing the ICP dry etching method, resulting in the sensing beam structure.

**Figure 10:**
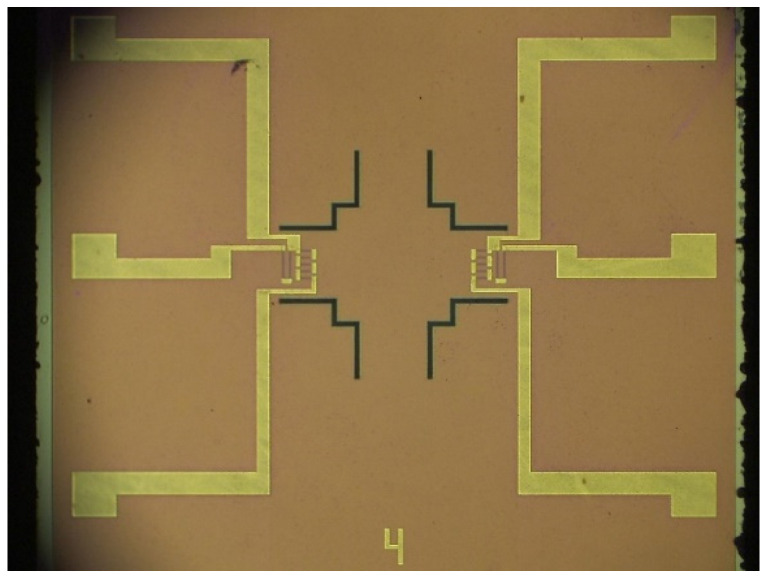
Chips under the microscope.

The finished sensor chip is shown in Figure 10, and the size of a single chip was 3 mm × 3 mm × 0.85 mm.

### 3.2. Improved Reliable Electrodes

To determine a suitable metal material for the ohmic contact, four metals (Pt, Ni, Ti, and Al) were tested in the ohmic contact area. Initially, we assessed whether these metals could form ohmic contacts with p-type silicon. The I–V characteristic curve of each resistor was measured using a semiconductor analyzer. The test was conducted in two stages: direct measurement immediately after sputtering and a second measurement after resting for one week.

**Figure 11:**
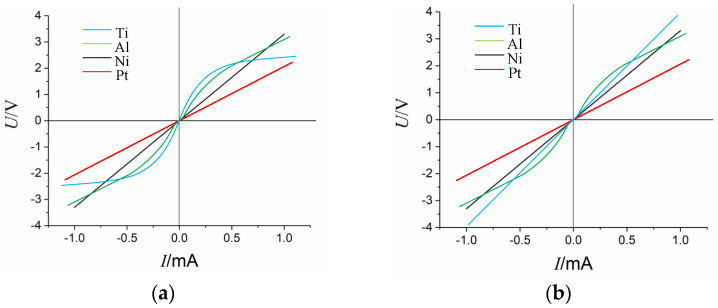
Types of I–V curves for four metals forming metal–semiconductor contacts: (**a**) directly after sputtering; (**b**) after one week of resting.

The test results are illustrated in Figure 11. When tested immediately after sputtering, the I–V curves of the Pt and Ni contacts with p-type silicon exhibited linearity, indicating the formation of ohmic contacts. Conversely, the I–V curves of the Ti and Al contacts with p–type silicon displayed nonlinearity, indicating the formation of rectifying contacts. Notably, the contact types established by the Pt, Ni, and Al metals with p-type silicon remained essentially unchanged before and after a one-week resting period, with only the Ti contact undergoing alteration. Initially, the I–V curves of the Ti metal contact were nonlinear, indicating rectifying contact. However, after resting for one week, the I–V curves became linear, indicating a transition to an ohmic contact.

Due to the propensity of both Ti and Si to oxidize, when Ti is sputtered onto the ohmic contact area through the magnetron sputtering process, the initial contact between Ti and Si involves pure physical contact. However, the presence of oxide on the contact surface creates a barrier, maintaining the nonlinearity of the I–V curve. Over the one week, Ti and Si underwent diffusion, resulting in the formation of titanium–silicon compounds. Consequently, the potential barrier at the contact surface decreased, and the p–type silicon in the contact area became integrated with the Ti–Si compound. Because the contact region of p-type silicon is heavily doped, the barrier layer on the contact surface becomes extremely thin, allowing electrons to pass through via tunneling effects, thereby resulting in an ohmic contact. On the other hand, the work function of Al is significantly lower than that of p–type silicon, and aluminum readily oxidizes to form alumina. Therefore, it is anticipated that aluminum and heavily doped silicon cannot form an ohmic contact under normal conditions.

Heating experiments were conducted on the ohmic contacts formed by Ti–Pt–Ni metals to test their resistance stability. Pt demonstrated strong thermal stability, with minimal changes in its resistance value immediately after the high-temperature stability test.

After analyzing the experiments above, it was concluded that Pt is the most suitable metal for the ohmic contact area. However, considering Pt’s poor adhesion to silicon dioxide, only a Pt layer was used to cover the ohmic contact area to ensure proper contact. For the lead layer, Ti–Pt–Au multilayer metal lead technology was employed. The Ti layer serves as an adhesive layer with strong adhesion to the silicon dioxide insulating layer and also fully envelopes the Pt layer, addressing Pt’s adhesion problem. Additionally, Au, chosen for its good conductivity, was used as the uppermost layer of metal. This arrangement facilitates the transfer of signals from the gold wire lead to the PCB board, ultimately completing the Pt–Ti–Pt–Au multilayer ohmic contact and metal leads.

### 3.3. Package

**Figure 12:**
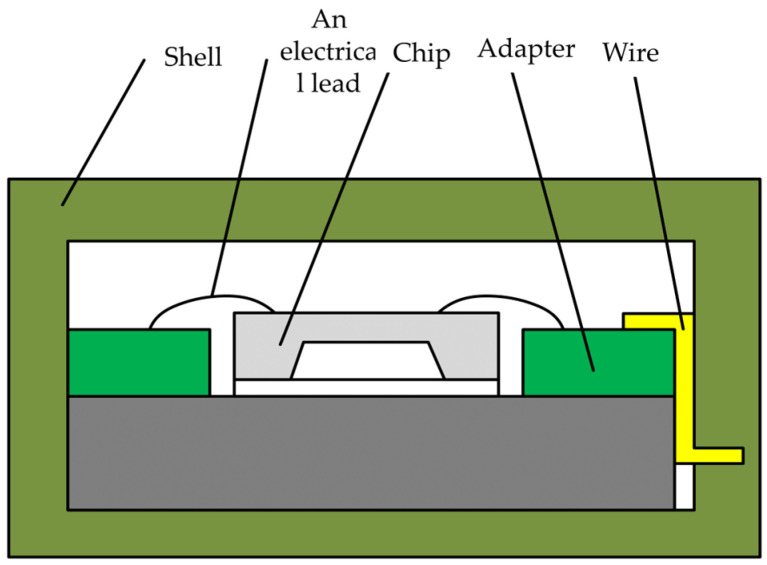
The packaging scheme.

To ensure the proper functioning of the accelerometer, the chip must be packaged. The packaging shell was made of stainless steel. The adhesive used for attaching the chip and transfer circuit board was applied to the bottom of the metal shell base. Electrical signals were interconnected between the chip and the circuit board via bonding wires. The other end of the circuit board outputted the measurement signal. Finally, the metal shell cover plate was welded to create a sealed enclosure. The packaged accelerometer is shown in Figure 12.

## 4. Testing and Analysis

The resistance test results indicated that the piezoresistor’s resistance value ranged between 3.3 kΩ and 3.4 kΩ, which closely matches the planned resistance value of 3.2 kΩ. The minimal variance in resistance among the sensors highlights the stability of the ion-implanted varistor. This stability ensures a minimal zero deviation when forming the Wheatstone bridge, making it easier to achieve zero balance through external circuit adjustments. The sensor with the smallest resistance error can be selected for subsequent experiments.

### 4.1. Hammering Test

**Figure 13:**
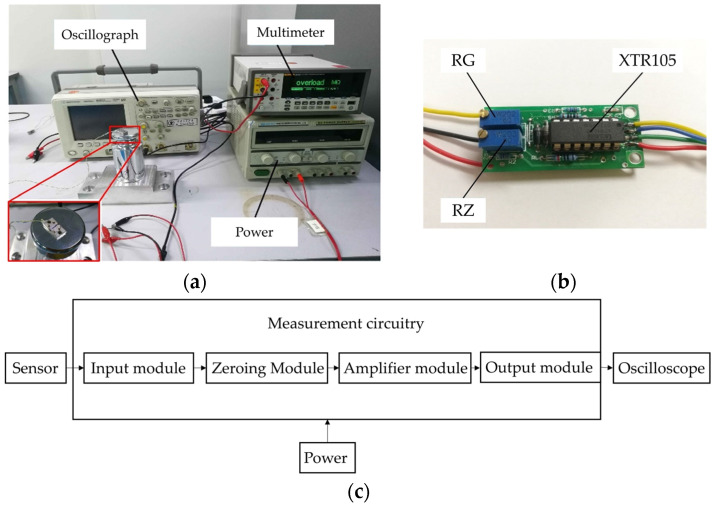
Sensor hammer test: (**a**) hammering test bench; (**b**) test boards; (**c**) test circuit working block diagram.

The hammering test was used to preliminarily assess the sensor’s operational capability and response to acceleration impacts. A hammer test setup, shown in Figure 13a, includes modules such as a sensor, power source, measurement circuitry, and oscilloscope. The test circuit board, depicted in Figure 13b, includes signal input, zero calibration, amplification, and signal output modules. This setup uses two potentiometers to adjust the bridge’s zero balance and amplification. The XTR105 chip converts the Wheatstone bridge’s differential signal into a voltage output and supplies a constant 1.5 mA current to the bridge. The entire hammer test workflow is outlined in Figure 13c.

First, the entire test circuit line connection is completed, and the sensor test circuit zero is adjusted, and then the sensor is fixed on the hammer surface; the hammer strikes the workbench vertically. The differential signal is input to the measurement circuit board after adjusting the amplification of the signal through the voltage form of the output. An oscilloscope is used to collect and display the voltage signal using the measurement circuit board through the 24 V constant voltage power supply.

**Figure 14:**
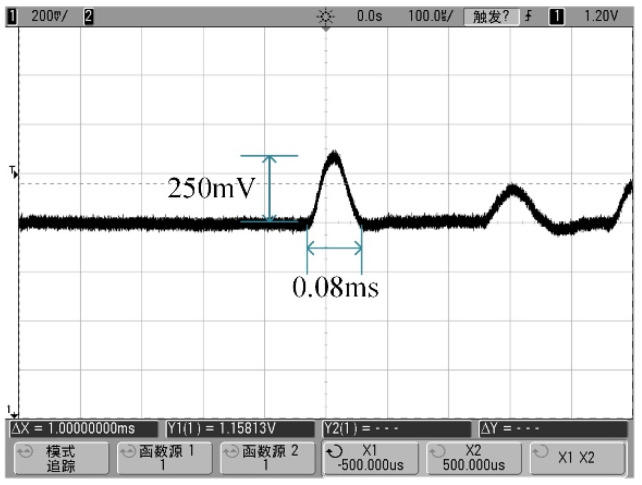
Hammering test output signal (250 mV is the amplified output signal, and 0.08 ms is the duration).

During the hammering experiment, the produced acceleration signal was minor. Therefore, a 50× amplification was applied to facilitate the observations. As shown in Figure 14, the amplified signal reached around 250 mV, corresponding to an actual output of approximately 5 mV over a 0.08 ms duration. Given that the hammering-induced acceleration on the worktable surface was only a few thousand g, the sensor’s output sensitivity was preliminarily estimated to be between 0.5 and 2 μV/g. This estimation served as a benchmark for the subsequent adjustments in the measurement circuit for other tests.

### 4.2. Impact Table Calibration Test

The commonly used test equipment for calibrating high-g accelerometers includes high acceleration shock test rigs and Marshallite hammer test rigs [16]. The sensor’s calibration utilized the SY13A-1 high acceleration (Dongling Vibration Experimental Instrument Company, Suzhou, China) impact test bench, with the signal amplification set to a 20-fold increase. The impact body was elevated to heights of 200 mm, 250 mm, 300 mm, 350 mm, and 400 mm. Notably, the sensor’s output signal exhibited a delay after the impact rather than quickly returning to zero. To ensure test data accuracy, there was a time gap after each impact test to allow the sensor’s zero balance to auto-correct. For every impact height, the output signal was recorded five times, and an average value was calculated.

The output voltage values for multiple measurements at different heights are shown in Table 1.

**Figure 15:**
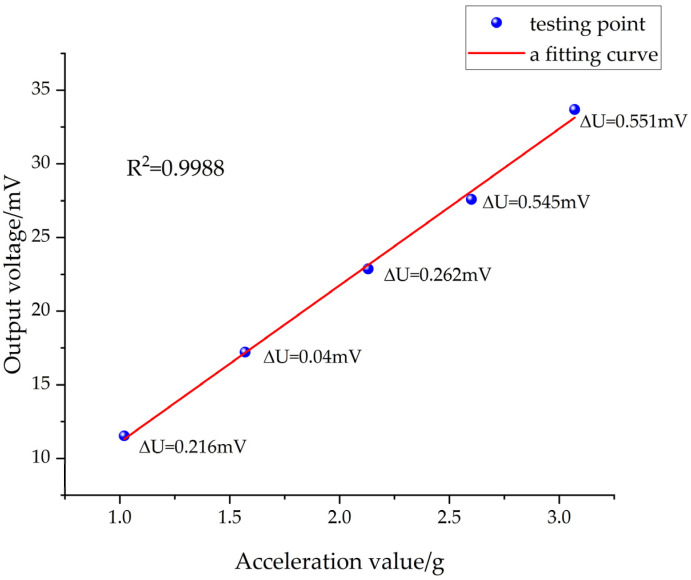
Sensor output voltage fitting curve.

After determining the true output voltages of the sensors at different values of impact acceleration, the data in Table 1 were least-squares fitted, and the fitted curve is shown in Figure 15, which is expressed as

(17) $$U_{o}=1.06\times 10^{-3}a+0.4555$$

where *U_o_* is the real output voltage (mV) and *a* is the acceleration value (g).

The original data points for the sensor’s output signal were evenly scattered around the fitting curve. The close proximity and linearity of this curve indicate the sensor’s high linearity. The fitting curve equation showed that the sensor’s output sensitivity was approximately 1.06 μV/g under a 1.5 mA constant current supply.

The ratio between the greatest difference between the calibration curve and the fitted curve of the sensor and the full-scale output is known as the sensor linearity [17]. Given the sensor’s design range of 100,000 g, the full-scale output under a 1.5 mA constant current supply can be determined from the fitting curve. However, since the peak impact acceleration in the calibration experiments only reached 30,700 g, the full-scale output within this calibration range should be used when calculating the sensor’s linearity. The maximum deviation between the sensor’s calibration and fitting curves can be used to determine the sensor’s linearity [18]; the linearity of the sensor at the five test heights was 1.92%, 0.23%, 1.14%, 1.95%, and 1.67%, respectively. The largest of these errors is the linearity of the sensor:

(18) $$\delta_{L}=\frac{\Delta U}{U_{o}}=\frac{0.545mV}{28.0155mV}=1.95\%$$

where *δ_L_* is the sensor linearity; $U_{o}$ is the theoretical output of the test points on the fitted line; and Δ*U* is the difference between the test point and fitted curve.

**Figure 16:**
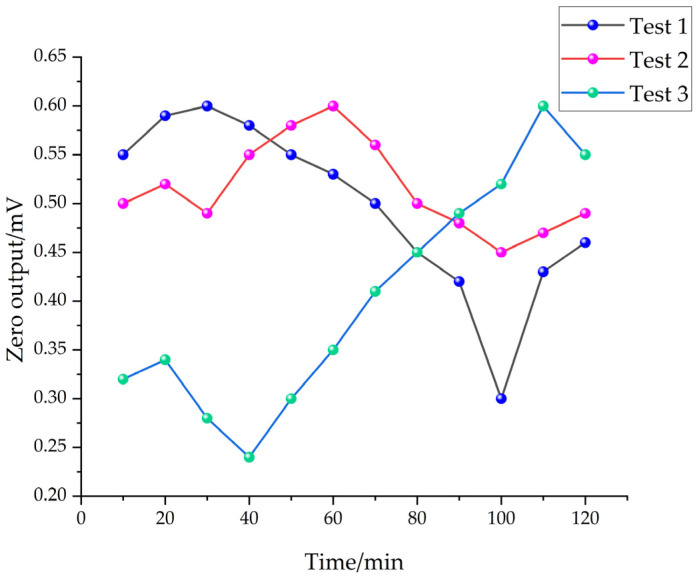
Zero drift of high g-value accelerometer.

To assess the zero drift of high-g accelerometers, many experiments were conducted over several days at an ambient temperature of 25 °C. The results are depicted in Figure 16. Under the given conditions and using Equation (19), the sensor’s zero drift was determined to be 0.29%. The absolute error of zero drift was approximately 146.2 g. This low drift value indicates that the sensor was stable and its output remained consistent under regular operating conditions.

(19) $$\delta_{D}=\frac{\Delta U_{0\max}}{U_{oF}}=\frac{0.31mV}{106mV}=0.29\%$$

where *δ_D_* is the zero drift; Δ*U*_0*max*_ is the zero output maximum difference; and *U_oF_* is the full-scale output.

### 4.3. Hopkinson Bar Test

**Figure 17:**
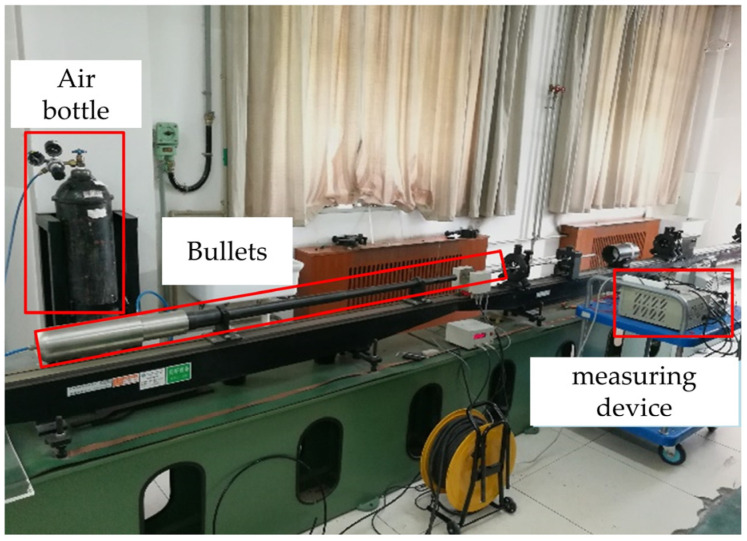
Hopkinson bar test.

To investigate the dynamic properties of the shock accelerometer, the Hopkinson pressure bar was used, which is capable of generating short-duration acceleration loads of up to 100,000 g [19], as shown in Figure 17. The signal acquisition device of the transducer was the same as that of the hammering test, and the air pressure of the air gun was first adjusted to 0.2 MPa during the test. The acceleration value of the incident bar under this air pressure was approximately 100,000 g.

**Figure 18:**
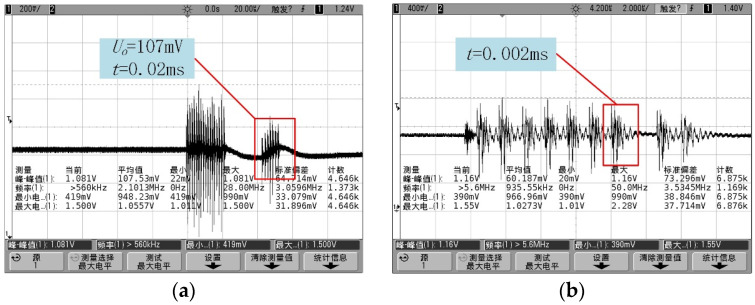
Hopkinson rod test signal output: (**a**) 100,000 g acceleration impact (107 mV is the output voltage, 20 μs is the duration of a single pulse.); (**b**) 150,000 g acceleration impact (2 μs is the signal pulse width).

The output voltage signal of the sensor, which was about 107 mV, is shown in Figure 18a, which corresponds to a full-scale output of 106.92 mV for the calibrated sensor sensitivity. The acceleration signal’s single pulse duration was around 20 μs, indicating that the sensor operated above 50 kHz. When the air gun pressure was adjusted to 0.3 MPa, producing an acceleration signal of 150,000 g on the incident bar, the sensor’s output (depicted in Figure 18b) showed a pulse width of 2 μs, corresponding to a frequency above 500 kHz. This high frequency exceeds the measurement capacity of the current sensor and testing circuit, but the output waveforms confirmed that the sensor was undamaged and could return to zero balance after a 150,000 g acceleration impact.

After the resistance test, hammering experiment, impact table calibration test, and Hopkinson rod test, some of the performance parameters of the composite beam structure high-g value accelerometer designed in this paper were calibrated, and the calibration values are shown in Table 2.

## 5. Conclusions

This study proposed a high-g value and high-overload acceleration sensor tailored for invasive weapons. We presented a novel composite beam structure that deviates from the traditional mass block type. Calibration tests on the impact table revealed a sensor sensitivity of 1.06 μV/g and a linearity of 1.95%, showcasing the sensor’s stability. The results from the Hopkinson rod test affirmed that the sensor operates efficiently up to its maximum range of 100,000 *g* and can endure acceleration impacts up to 150,000 *g* without damage, indicating its significant overload capacity.

## Figures and Tables

**Table 1:** Real voltage output of the sensor under different acceleration impacts.

Impact Acceleration × 10^4^ (g)	Accelerometer Output Voltage (mV)					
	First	Second	Third	Fourth	Fifth	Average
1.02	13.02	11.44	12.16	10.22	10.84	11.53
1.57	16.82	18.04	16.52	18.20	16.46	17.21
2.13	23.68	22.06	22.88	23.18	22.56	22.87
2.60	27.14	28.28	28.16	27.38	26.98	27.59
3.07	32.92	34.38	35.18	35.48	30.48	33.69

**Table 2:** Sensor performance indicators.

	Zero Drift	Sensitivity	Linearity	Range	Overload
Value	0.29%	1.06/μV∙g^−1^	1.95%	100,000/g	>150,000/g

## Data Availability

Data are contained within the article.
